# Supplementary material for: Infection and Coinfection of Porcine-Selected Viruses (PPV1 to PPV8, PCV2 to PCV4, and PRRSV) in Gilts and Their Associations with Reproductive Performance
Source: Vet Sci. 2024 Apr 24;11(5):185. doi: 10.3390/vetsci11050185 (PMC11125912; doi:10.3390/vetsci11050185)
Supplement: Supplementary file 1 [file vetsci-11-00185-s001.zip › vetsci-2914885-supplementary.pdf]

Table S1. Primers and probes used in the present study for the detection of PPV1 through PPV7, PCV2, PCV3, and PRRSV through real-time PCR

Primer/Probe	Nucleotide sequence (5´-3´)	Amplicon	Genbank position/Access number	Reference
PCV2abF<br>PCV2abR<br>ProbePCV2a<br>ProbePCV2b/d	GCAGGGCCAGAATTCAACC<br>GGCGGTGGACATGATGAGA<br>FAM-GGGGACCAACAAAATCTCTATACCCTTT-BHQ<br>Cy5-CTCAAACCCCCGCTCTGTGCCC-BHQ	123	1411-1534/MZ747121a	[48]
PCV3F<br>PCV3R<br>Probe PCV3	AGACGACCCTTATGCGGAAA<br>AACGGTGGGGTCATATGTGTTG<br>FAM-CTCACCCAGGACAAAGCCTCTTCTT-BHQ	155	1448-1603/OL799306 b	[49]
PPV1F<br>PPV1R<br>Probe PPV1	GAAGACTGGATGATGACAGATCCA<br>TGCTGTTTTTGTTCTTGCTAGAGTAA<br>FAM-AATGATGGCTCAAACCGGAGGAGA-BHQ	120	1188-1308 KF913350c	[47]
PRRSVF<br>PRRSVR<br>Probe PRRVS	GTAGTYGCRCTCCTTTGGGGRGTGT<br>GACGCCGRACGASAAAYGCGTGGTTA<br>FAM-TACATTCTGGCCCCTGCCCAYC-TAMRA	173	14607-/MN642104d	[46]
PPV2F<br>PPV2R	AGCTCTGCGACAAGTGGG<br>GTCTACGGCCTGCAAGAA	186	235-421/MW051675e	[10]
PPV3F<br>PPV3R	CAYGAYGAACGGTACGATGAAAT<br>GCGGTAAAACCTGTGAWAWTTGAAC	222	2932-3154/ JF738367 f	[41]
PPV4F<br>PPV4R	TATGTGGGCTGGGCAAGGAATGTC<br>GTTGCGGAATGCTATCAGGCTCTT	416	1068-1771/GQ387500i	[24]
PPV5F<br>PPV5R	GCATTGGTGTGTGTCTGTGTCC<br>GTGGCACATTTGTACATGGGAG	344	1068-1412/JX896321j	[24]
PPV6F<br>PPV6R	GGCTTCATAATCCCTCCAAAACCT<br>GCTCATCTTCCTCTTGTTTCTCCTG	156	3369-3525/MW051672k	[42]
PPV7F<br>PPV7R	GAGGCGGTGATGGAGCAGAT<br>CTCCAGGACCACCACATCCC	119	1170/MN326293.1l	[43]

[a-k] Gen Bank reference sequence of each virus.

Table S2. Percent of positive farms and samples prevalence by real-time for PPVs (PPV1 through PPV7), PCV2, PCV3, and PRRSV.

Virus	% Positive farms (positive / all tested)	% Positive sera (positive / all tested)
**PRRSV**	82.5 (33 / 40)	63.6 (149 / 234)
**PCV2**	85 (34 / 40)	53.4 (125 / 234)
**PCV3**	57.5 (23 / 40)	30.7 (72 / 234)
**PPV1**	17.5 (7 / 40)	14.5 (34 / 234)
**PPV2**	22.5 (9 / 40)	9.8 (23 / 234)
**PPV3**	67.5 (27 / 40)	40.1 (94 / 234)
**PPV4**	15 (6 / 40)	4.2 (10 / 234)
**PPV5**	40 (16 / 40)	20.5 (48 / 234)
**PPV6**	32.5 (13 / 40)	17 (40 / 234)
**PPV7**	5 (2 / 40)	1.28 (3 / 234)

Table S3. Viral infection and coinfections in gilts at the herd level from 40 herds in Colombia.

Detection status	Number of herds	Viral detection (number of herds)
Single	0	-
Double	3	PCV2/PRRSV (1), PCV2/PPV6 (1), PPV1/PPV3 (1).
Triple	9	PCV2/PRRSV/PPV1 (2), PCV2/PPV5/PRRSV (2), PCV3/PRRSV/PPV1 (1), PCV3/PCV2/PRRSV (1), PCV2/PPV2/PRRSV (1), PPV2/PPV3/PRRSV (1), PCV2/PPV3/PRRSV (1).
Quadruple	10	PCV2/PCV3/PRRSV/PPV3 (1), PCV2/PCV3/PPV1/PPV3 (1), PCV2/PCV3/PPV1/PPV5 (1), PCV2/PCV3/PPV2/PPV3 (1), PCV2/PCV3/PPV3/PPV6 (1), PCV2/PRRSV/PPV1/PPV5 (1), PCV2/PRRSV/PPV3/PPV5 (1), PCV3/PRRSV/PPV3/PPV5 (1), PCV3/PRRSV/PPV3/PPV6 (1), PPV3/PPV4/PPV5/PPV6 (1).
Quintuple	9	PCV2/PCV3/PRRSV/PPV1/PPV2 (1), PCV2/PCV3/PRRSV/PPV1/PPV3 (1), PCV2/PCV3/PRRSV/PPV1/PPV6 (1), PCV2/PCV3/PRRSV/PPV3/PPV5 (1), PCV2/PCV3/PRRSV/PPV3/PPV6 (1), PCV2/PRRSV/PPV1/PPV3/PPV5 (1), PCV2/PRRSV/PPV3/PPV5/PPV6 (1), PCV2/PRRSV/PPV3/PPV4+PPV5 (1), PCV3/PRRSV/PPV3/PPV5/PPV6 (1).
Sextuple	6	PCV2/PCV3/PRRSV/PPV1/PPV2/PPV3 (2), PCV2/PCV3/PRRSV/PPV1/PPV3/PPV4 (1), PCV2/PCV3/PRRSV/PPV1/PPV3/PPV5 (1), PCV2/PCV3/PRRSV/PPV3/PPV5/PPV6 (1), PCV2/PCV3/PRRSV/PPV2/PPV3+PPV5 (1).
Septuple	2	PCV2/PCV3/PRRSV/PPV3/PPV4/PPV5/PPV6 (1), PCV2/PRRSV/PPV2/PPV3/PPV4/PPV6/PPV7 (1).
Octuple	1	PCV2/PCV3/PRRSV/PPV1/PPV3/PPV4/PPV6/PPV7 (1).

Table S4. Viral infection and coinfections detected in gilts from 40 herds in Colombia.

Infection status	Number of sera	Viral coinfection (number of sera)
No infection	0	-
Single infection	39	PRRSV (14), PPV3 (9), PCV2 (7), PCV3 (4), PPV5 (3) PPV1 (2).
Double infection	69	PCV2/PRRSV (21), PCV2/PPV6 (7), PCV2/PCV3 (6), PRRSV/PPV1 (6), PRRSV/PPV3 (6), PPV1/PPV3 (4), PRRSV/PPV5 (3), PCV3/PRRSV (2), PCV2/PPV1 (2), PCV2/PPV3 (2), PCV2/PPV5 (2), PCV3/PPV2 (2), PPV3/PPV6 (2), PCV2/PPV4 (1), PCV3/PPV3 (1), PPV2/PPV3 (1), PPV3/PPV5 (1).
Triple infection	64	PCV2/PRRSV/PPV3 (9), PCV2/PRRSV/PPV5 (9), PCV2/PCV3/PRRSV (5), PCV3/PRRSV/PPV3 (6), PCV2/PCV3/PPV1 (4), PCV2/PRRSV/PPV2 (4), PRRSV/PPV2/PPV3 (4), PRRSV/PPV3/PPV5 (4), PCV2/PRRSV/PPV1 (3), PCV2/PPV3/PPV6 (3), PCV3/PRRSV/PPV6 (2), PCV2/PCV3/PPV2 (1), PCV2/PCV3/PPV3 (1), PCV2/PCV3/PPV5 (1), PCV2/PRRSV/PPV6 (1), PCV2/PPV1/PPV5 (1), PCV2/PPV3/PPV5 (1), PCV3/PRRSV/PPV2 (1), PCV3/PRRSV/PPV5 (1), PCV3/PPV1/PPV3 (1), PCV3/PPV2/PPV3 (1), PCV3/PPV3/PPV5 (1), PCV3/PPV3/PPV6 (1).
Quadruple infection	36	PCV3/PRRSV/PPV3/PPV6 (9), PCV2/ PRRSV/PPV3/PPV5 (6), PCV2/PCV3/PRRSV/PPV3 (3), PCV2/PCV3/PRRSV/PPV5 (3), PCV2/PCV3/PRRSV//PPV6 (2), PRRSV/PPV1/PPV3/PPV6 (2), PCV2/PCV3/PRRSV/PPV1 (1), PCV2/ PRRSV/PPV1/PPV5 (1), PCV2/ PRRSV/PPV1/PPV6 (1), PCV2/PRRSV/PPV2/PPV3 (1), PCV2/PRRSV/PPV3/PPV4 (1), PCV2/PRRSV/PPV4/PPV6 (1), PCV3/PRRSV/PPV1/PPV5 (1), PCV3/PPV1/PPV3/PPV4 (1), PRRSV/PPV4/PPV5/PPV6 (1), PPV3/PPV4/PPV5/PPV6 (1).
Quintuple infection	12	PCV2/PCV3/PRRSV/PPV3/PPV6 (2), PCV2/PCV3/PRRSV/PPV5/PPV6 (2), PCV2/PRRSV/PPV1/PPV3/PPV4 (1), PCV2/PRRSV/PPV1/PPV3/PPV5 (1), PCV2/PRRSV/PPV1/PPV3/PPV6 (1), PCV2/PRRSV/PPV2/PPV3/PPV4 (1), PCV2/PRRSV/PPV4/PPV6/PPV7 (1), PCV3/PRRSV/PPV1/PPV3/PPV6 (1), PCV3/PRRSV/PPV2/PPV3/PPV5 (2), PCV3/PRRSV/PPV3/PPV5/PPV6 (1).
Sextuple infection	3	PCV2/PCV3/PRRSV/PPV1/PPV3/PPV6 (1), PCV2/PCV3/PRRSV/PPV2/PPV3/PPV5 (1), PCV2/PRRSV/PPV3/PPV4/PPV6/PPV7 (1)
Septuple infection	1	PCV2/PCV3/PRRSV/PPV1/PPV3/PPV6/PPV7 (1).
